# Multipass Active Contours for an Adaptive Contour Map

**DOI:** 10.3390/s130303724

**Published:** 2013-03-15

**Authors:** Jeong Heon Kim, Bo-Young Park, Farhan Akram, Byung-Woo Hong, Kwang Nam Choi

**Affiliations:** Department of Computer Science & Engineering, Chung-Ang University, Seoul 156-756, Korea; E-Mails: jhkim@vim.cau.ac.kr (J.H.K.); parkby@image.cau.ac.kr (B.-Y.P.); farhan@vim.cau.ac.kr (F.A.); hong@cau.ac.kr (B.-W.H.)

**Keywords:** biomedical image processing, active contours, level sets, contour map, Mumford-Shah energy functional, level set evolution without re-initialization, initial contour problem, local optimum problem

## Abstract

Isocontour mapping is efficient for extracting meaningful information from a biomedical image in a topographic analysis. Isocontour extraction from real world medical images is difficult due to noise and other factors. As such, adaptive selection of contour generation parameters is needed. This paper proposes an algorithm for generating an adaptive contour map that is spatially adjusted. It is based on the modified active contour model, which imposes successive spatial constraints on the image domain. The adaptability of the proposed algorithm is governed by the energy term of the model. This work focuses on mammograms and the analysis of their intensity. Our algorithm employs the Mumford-Shah energy functional, which considers an image's intensity distribution. In mammograms, the brighter regions generally contain significant information. Our approach exploits this characteristic to address the initialization and local optimum problems of the active contour model. Our algorithm starts from the darkest region; therefore, local optima encountered during the evolution of contours are populated in less important regions, and the important brighter regions are reserved for later stages. For an unrestricted initial contour, our algorithm adopts an existing technique without re-initialization. To assess its effectiveness and robustness, the proposed algorithm was tested on a set of mammograms.

## 1. Introduction

The extraction of meaningful information from images by means of digital image processing techniques is an important task in many application domains. To identify significant information in an image, one can exploit distinctive features of objects (e.g., shape, location, margin) and uncover significant regions or patterns by analyzing the topological and geometrical properties of the image. In the biomedical sector, medical imaging techniques, such as X-rays, magnetic resonance imaging (MRI), and tomography are used to visualize internal structures of the body. Medical images, such as mammograms, have inherently complex and variable features with blurred object boundaries, which make the use of explicit features of objects in image analysis difficult. Hence, image analysis methods based on isocontour mapping are better suited to complex medical images as mentioned below.

Meaningful information can be extracted efficiently from a digital image on an isocontour map. In biomedical image processing, isocontour mapping is used extensively to perform the topographic analysis of medical images. An isocontour map consists of a set of curves of equal value (e.g., height or intensity). Analyses based on isocontour maps can provide the association between image inclusions. They detect a region of interest (ROI) by analyzing the correlation (or the enclosure relationship) between objects. ROI analysis that highlights suspicious regions in medical images is an essential step in computer-aided diagnosis systems. Thus, isocontour maps can provide a robust topographic representation of medical images for ROI analysis [1].

To our current knowledge there is no deterministic way to find parameters like the number of quantization levels (contour interval and the difference in elevation between successive contours) that yield the best results for isocontour generation. However, the determination of contour generation parameters is an open question and adjustable. Hong and Sohn [1] proposed a multiscale approach for ROI segmentation, which extracts isocontours at multiple scales and analyzes mammographic features in a hierarchical manner from a coarse scale to a fine one. This multiscale approach was necessary because the information provided by isocontour maps with fixed parameters was sometimes either too excessive or scarce due to varying image conditions. This paper aims to produce an adaptive contour map that provides “not too much and not too little” information by adapting active contours spatially during the curve evolution.

A number of active contour models have been developed. Kass *et al.* [2] proposed a successful method based on variational and partial differential equations (PDE), the well known active contour/snake model, to extract interesting objects in an image. Various active contour models and enhanced versions are employed in various image processing applications, as well as medical images. The active contours are represented as parameterized curves in a Lagrangian framework [2] and the implicit curves are given in an Eulerian framework [3–6].

Geodesic active contour (GAC) models in [3,4] are geometrically intrinsic and embed the level set function [7], which involves the representation of the implicit curve. The curve evolution with the level set function naturally splits and merges the contours during the evolution, and therefore automatically handles topological changes. The curves evolve based on the minimization of the energy functional from the image, the curve, and the level set function. Energy functionals are used in the energy of edge-based model [2–5] and the region-based model [6].

The classical active contour model [2–8], which detects objects in an image, starts with a given initial contour and performs the curve evolution to find the optimal contour. In the algorithm for adaptive contour mapping proposed in this paper, the initial contour divides the image domain into sub-regions in which a new optimal contour is found. In subsequent iterations, the contour would have a different spatial domain from that of the previous contour. This domain segmentation (or the curve evolution) is repeated until the stopping criterion is met, thereby creating an adaptive contour map of the image. The adaptability of the proposed algorithm is governed by the energy term of the active contour model, so it is important to employ one that is both effective and reliable.

The proposed algorithm for adaptive contour mapping is based on two previous active contour models: active contours without edges (ACWE) and level set evolution without re-initialization (LSEWR). From the ACWE concept, we used force image terms to get regional information, whereas in LSEWR we selected penalizing terms to eliminate re-initialization. The ACWE model proposed by Chan and Vese [6] considers the intensity distribution of an image to establish an optimality criterion for segmenting the image into sub-regions; therefore, it is suitable for use in analyzing mammographic intensities. The ACWE model finds an optimal partition from the energy of a region in an image that has a weak edge and heavy noise. The ACWE method converges relatively faster than edge based active contours [2–5] because the merging of similar regions occurs broadly while contours move narrowly. The level set function partitions a region into an inside and outside of a zero level curve. An extension of the ACWE model is proposed for a multi-phase segmentation. Vese and Chan [8] proposed the multi-phase segmentation model with n level set functions. This method always presents 2^n^ regions from the combination of each phase by level set functions. The curve evolution with the level set function requires costly re-initialization because the level set function deviates from a signed distance function (SDF) in each evolution. Li *et al.* [5] proposed the LSEWR model, which consists of an internal energy term that penalizes the deviation of the level set function from an SDF, and thus eliminates re-initialization.

Our algorithm is designed with a similar manner of isocontour mapping to detect an arbitrary number of contours for spatial adaptive isocontour mapping. The existing multi-phase method, which detects contours at multiple level sets, always produces 2^n^ regions. This indicates that many insignificant features might be included in the contour map, thereby influencing the image analysis results. Our approach divides a region into two sub-regions using the base contour. It then divides one of the segmented sub-regions into two sub-regions in successive iterations. The proposed algorithm detects sub-regions by minimizing the new energy model, restricting it to the characteristic function of a base sub-region. The iterative segmentation process automatically terminates when the stopping criterion is met. Note that only one of the two sub-regions is further segmented in successive iterations. This is associated with the characteristics of the mammographic image in addition to problems in initialization and local optimum of the active contour model.

In mammograms, bright regions contain information that is more significant (e.g., candidate masses). Our algorithm takes advantage of this mammographic characteristic to address the problems in initialization and local optimum of the active contour model. That is, the proposed algorithm starts with the initial contour found in the darkest (low intensity) region so that the local optima encountered during the contour evolution is placed in less important low intensity regions. In terms of initialization, this enables the our approach to start its operation with an initial contour in the darkest region of the image, and the LSEWR component allows free initial contour without satisfying SDF. Hence, the important brighter regions are kept for segmentation and refined analysis at later stages.

**Figure 1.**
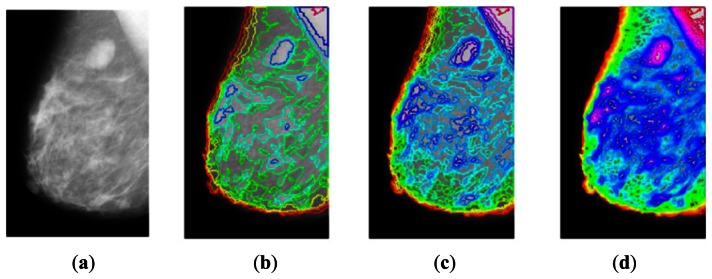
Multiscale approach for isocontour maps [1]: (**a**) Mammogram, (**b**) Coarsescale isocontour map, (**c**) Intermediate-scale isocontour map, (**d**) Fine-scale isocontour map.

In this paper, we propose an adaptive contour mapping approach that can be used to analyze the topological and geometrical information on mammogram images. Mammograms have complex and variable structures with blurred object boundaries, which make the use of explicit object features that are less applicable to image analysis. Hence, this work utilizes an isocontour map to analyze mammographic features. It is relatively easy to create an isocontour map, but the parameters for generating active contours should be determined accordingly. As shown in Figure 1, the existing multiscale approach [1] extracts isocontours at multiple scales and analyzes mammographic features in a hierarchical manner from a coarse scale to a fine one. The proposed spatially adaptive contour map conveys the topographic information of a mammogram with an arbitrary number of contours, which reduces the complexity of analytical methods based on isocontour map images. We also develop an algorithm for adaptive contour mapping, the multipass active contour approach, which is based on the ACWE and LSEWR models. The proposed algorithm addresses the initialization and local optimum problems inherent in the classical active contour model by using the characteristics of mammograms (*i.e.*, significant information is located in brighter regions). Finally, our algorithm makes the adaptive contour map with the spatial skip of isocontours which has similar energy by the energy term of the algorithm.

The rest of the paper is organized as follows: we discuss related work in Section 2. In Section 3, we discuss the proposed multipass active contour approach. In particular, our algorithm is designed to partition the image domain into an arbitrary number of sub-regions by applying a two-phase segmentation algorithm based on the recursive use of the Mumford-Shah energy functional. The recursive application of the two-phase segmentation algorithm on subsequently segmented sub-regions results in a tree structure of partitions for an adaptive contour map. In Section 4, we discuss denoising of the input image, numerical scheme, initialization of the level set function, and the parameters needed to implement the algorithm. We then describe the data set and the analytical results in Section 5. We finish our contribution with several conclusions.

## 2. Active Contours

Active contours are a core component of computer vision and medical imaging, and they can be divided into three areas: boundary driven, region driven, and hybrid contours that combine the boundary and region driven areas.

Malladi *et al.* [4] presented an approach to shape modeling in which a speed term synthesized from the image is used to stop the contour near object boundaries. They used geometric flows for boundary extraction in which distance transforms were used as embedding functions. Caselles *et al.* [3] proposed a geodesic approach for object segmentation, which allows connecting classical “snakes” based on energy minimization and geometric active contours based on the theory of curve evolution. Paragios *et al.* [9] proposed an edge-driven bidirectional geometric flow for boundary extraction. They combined the geodesic active contour flow and the gradient vector flow external force for snakes. The resulting motion equation is considered within a level set formulation that can deal with topological changes and important shape deformations. Weickert *et al.* [10] presented fast algorithms based on the semi-implicit additive operator splitting (AOS) scheme for both geometric and geodesic active contour models.

In 2001, Chan and Vese [6] proposed a new model for active contours to detect objects in a given image, based on the techniques of curve evolution, Mumford–Shah functional for segmentation, and level sets. Their model can detect objects whose boundaries are not necessarily defined by gradients. We minimize an energy that represents a particular case of the minimal partition problem. In the level set formulation, the minimal partition problem becomes evolving the active contour, which will stop at the desired boundary. However, unlike the classical active contour models, the stopping term does not depend on the gradient of an image, but instead is related to its homogeneity. Paragios *et al.* [11] introduced a frame partition paradigm within the level set space, which can account for boundary and global region-driven information. In 2005, Li *et al.* [5] presented a new variational formulation for geometric active contours, which forces the level set function to be close to a signed distance function, thus eliminating the need for the costly re-initialization procedure.

Vese and Chan [8] proposed a new multi-phase level set framework for image segmentation using the Mumford and Shah model for application in piecewise constant and piecewise smooth optimal approximations. The proposed method is also a generalization of an active contour model without edge-based two-phase segmentation. They introduced classifications according to a combination of all level sets at a given pixel. Cremers *et al.* [12] presented a novel variational approach for segmenting the image plane into a set of regions of piecewise constant motion based on only two consecutive frames from an image sequence. They proposed the implementation of this functional using a multi-phase level set framework. Minimizing the functional with respect to its dynamic variables results in an evolution equation for a vector-valued level set function and an eigenvalue problem for motion vectors.

Our algorithm is performed in such a way that it partitions an image into two sub-regions. One of the sub-regions is then iteratively partitioned into two more sub-regions. The sub-regions are determined by the minimization of a new energy model restricted to a characteristic function of a sub-region, and no re-initialization is needed. Our method segments the image into any number of regions, and the process automatically terminates at the stationary solution. In this paper, the proposed approach is performed from the lowest intensity region to important high intensity regions, which are thus segmented in fine scale. Finally, the segmentation result provides an adaptive contour map of the image.

## 3. Proposed Multi-Pass Active Contours for an Adaptive Contour Map

A curve *C* in the image domain Ω is represented by the zero level set of a level set function *ϕ* : Ω → R. The curve *C* partition a k^th^ sub-region w_k_ ⊂ Ω into two sub-regions w_k+1_,w̅_k+1_ with, *ϕ* such that:

(1) $$\left\{ \begin{array}{l} \text{inside}(C)=w_{k+1}=\{x\in w_{k}:\phi (x)<0\},\\ \text{outside}(C)={\bar{w}}_{k+1}=\{x\in w_{k}:\phi (x)\geq 0\}. \end{array}\right.$$

**Figure 2.**
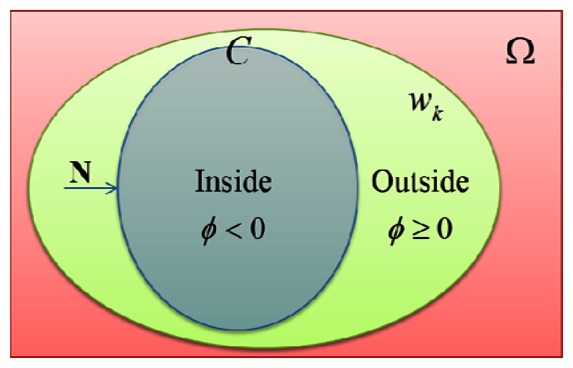
Partition by the curve *C* = {*x: ϕ*(*x*) =0} with the level set function *ϕ* restricted by a *k*^th^ sub-region *w_k_* in an image domain Ω with evolving of *C* to normal vector N.

Figure 2 illustrates the above assumptions and notations on the level set function *ϕ*, defining the evolving curve *C*. For the level set formulation of our variational active contour model, we replace the unknown variable *C* by the unknown variable where at the boundary of curve *C* the value of *ϕ* =0 and our level set function moves inward for further evolution.

**Figure 3.**
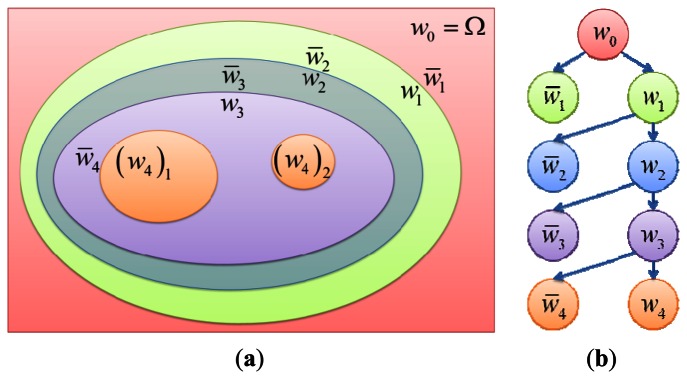
(**a**) Calculated adaptive contour map from a given image *w_0_*, (**b**) Inclusion tree from the adaptive contour map.

The region *W_k_* is iteratively re-partitioned into two sub-regions, and therefore the image domain has multiple segmented regions. Let us consider *w*_0_ as the input image, which is further segmented in two sub-regions *w*_1_ and w̅_1_, which are located inside and outside of the zero level set curve respectively. In our algorithm, the evolution of level set starts from outside the boundary of the given image and moves inwards. To move the level set inwards, we calculate the inner sub-region of the zero level curve using *ϕ* < 0. Figure 3(a) shows an image whose inner regions *w*_1_,*w*_2_,*w*_3_,*w*_4_ are calculated using *ϕ* < 0 and the outer regions are calculated by subtracting the energy components of the current calculated inner sub-region from the previously calculated inner region (or original image for the first outer component), respectively, as shown in the equations below:

(2) $${\bar{w}}_{k+1}=w_{k}-w_{k+1}$$

The isocontour of *w*_1_ and *w̅*_1_ is calculated from the region *w_0_*, which is the base sub-region as shown in Figure 3(b). The next contour is calculated from the previous inside sub-region *w*_1_ as shown in Figure 3(b). The next contour from *w*_1_ makes the next sub-regions *w*_2_ and *w̅*_2_. Further contours make further sub-regions *w*_3_,*w̅*_3_,*w*_4_ and *w̅*_4_.

The proposed segmentation algorithm can be expressed in the following iteration that the minimizer *ϕ* of an energy functional *E* becomes the parameter *φ^k^* of *E*:

(3) $$\varphi^{k+1}=\underset{\phi }{\arg\min}E\left(c_{1},c_{2},\phi ;\varphi^{k}\right)$$

where constants *c*_1_ and *c*_2_ depending on *ϕ* are means of denoised image *I* with respect to regions *w_k+_*_1_ and *w̅_k+_*_1_. If minimizer *ϕ* is equal to the initial of *ϕ*, the iteration terminates. We find the optimal partition from the minimizer *ϕ* of energy functional *E* with the sub-region *w_k_* depending on the parameter*φ^k^*. Our model of energy functional *E* is:

(4) $$E(c_{1},c_{2},\phi )=\lambda_{1}F_{1}(c_{1},\phi )+\lambda_{2}F_{2}(c_{2},\phi )+\mu L_{g}(\phi )+vA_{g}(\phi )+\alpha P(\phi )$$

where λ_1_, λ_2_ > 0, *μ v* ≥ 0, α > 0 are parameters, and:

*F*_1_ is energy of region;*w_k+1_*
*F*_2_ is energy of region *w̅_k+1_*;
*L*_g_ is weighted arc length of curve *C* by an edge indicator function;
*A*_g_ is weighted area of sub-region *w_k+_*_1_ by an edge indicator function;
*P* is penalizing energy in [5].

The energy functional *F*_1_,*F*_2_ would drive the motion of the zero level curve *C* of *ϕ*, *L*_g_ would regularize the curve *C*, and *A*_g_ would accelerate the curve evolution when all regions of *w_k_* and *P* would penalize the deviation of *ϕ* from a signed distance function (SDF). We introduce the region energy functional *F*_1_,*F*_2_ from the CV model [6] with the domain restriction, defined by:

(5) $$F_{1}(c_{1},\phi )=\int_{\Omega }{\left|I(x)-c_{1}\right|}^{2}H(-\phi (x))M^{k}(x)dx$$

(6) $$F_{2}(c_{2},\phi )=\int_{\Omega }{\left|I(x)-c_{2}\right|}^{2}H(\phi (x))M^{k}(x)dx$$

where *H* is Heaviside function and *M^k^* is a characteristic function of sub-region *w_k_*, such as:

(7) $$\left\{ \begin{array}{l} M^{k}(x)=H\left(-\varphi^{k}(x)\right)\\ M^{0}:\Omega \to 1 \end{array}\right.$$

The energy of sub-region *w_k_* only affects *E* by the region restriction function *M^k^*, which is calculated from *φ^k^*. The weighted arc length functional *L*_g_ with univariate Dirac Delta function *δ* = *H*′ in the level set method [7] is:

(8) $$L_{g}(\phi )=\int_{\Omega }g\delta (\phi )|\nabla \phi |dx$$

where the edge indicator function g is a positive and strict decreasing function. For example:

(9) $$g=\frac{1}{1+{\left|\nabla I\right|}^{2}}$$

The zero level curve *C* is driven into a smooth curve from a complicated curve to minimize the functional *L*_g_. By the level set method, the weighted area functional *A*_g_ is expressed as:

(10) $$A_{g}(\phi )=\int_{\Omega }gH(-\phi )dx$$

The small energy of *A*_g_ accelerates the curve evolution. By definition, an SDF satisfies a desirable property |∇ *I*|=1. Li *et al.* [5] proposed the functional:

(11) $$P(\phi )=\int_{\Omega }\frac{1}{2}{\left(|\nabla \phi |-1\right)}^{2}dx$$

as a metric. The functional *P* characterizes how close a function *ϕ* is to an SDF. The energy functional *A_g_* (*ϕ*) in Equation (10) is introduced to speed up the curve evolution. Note that when the function *g* is constant 1, the energy functional in Equation (10) is the area of the region $\Omega_{\phi }^{-}=\{x|\phi (x)<0\}$ [13]. The energy functional *A_g_* (*ϕ*) in Equation (10) can be viewed as the weighted area of $\Omega_{\phi }^{-}$. The coefficient *v* multiplying *A_g_* (*ϕ*) can be positive or negative, depending on the relative position of the initial contour to the object of interest. For example, if the initial contours are placed outside the object, the coefficient *v* in the weighted area term should take a positive value to allow the contours to shrink more quickly. If the initial contours are placed inside the object, the coefficient *v* should be negative to speed up the contours' expansion.

For the minimization, it is necessary to find the zero point of differentiation of the functional *E*(*c*_1_, *c*_2_, *ϕ*). By the calculus of variations [14], the Gateaux derivative (first variation) of the functional *E*(*c*_1_, *c*_2_, *ϕ*) in Equation (4) can be written as the gradient flow:

(12) $$\begin{array}{cc} -Q(\phi )= & -\delta (\phi )\left[\lambda_{1}{\left(I-c_{1}\right)}^{2}M^{k}-\lambda_{2}{\left(I-c_{2}\right)}^{2}M^{k}+\mu \text{div}\left(g\frac{\nabla \phi }{\left|\nabla \phi \right|}\right)+vg\right]\\ & -\alpha \left[\Delta \phi -\text{div}\left(\frac{\nabla \phi }{\left|\nabla \phi \right|}\right)\right]=0 \end{array}$$

where Δ is the Laplacian operator. Therefore, the function *ϕ*, which minimizes this functional, satisfies the Euler Lagrange equation ∂*E*/∂*ϕ =* 0. This gradient flow is the evolution equation of the level set function in the proposed method. The first and second terms on the right hand side of Equation (12) correspond to the energy functional *F*_1_ and *F*_2_, which partitions a region inside and outside of the zero curve *C* based on the energy functional values. The third and the fourth terms correspond to the gradient flows of the energy functionals *μ*L_g_(*ϕ*) and *vA_g_*(*ϕ*), respectively, and are responsible for driving the zero level curve towards the object boundaries. The fifth term, which is associated with the penalizing energy α*P*(*ϕ*), represents the gradient flow:

(13) $$\Delta \phi -\text{div}\left(\frac{\nabla \phi }{\left|\nabla \phi \right|}\right)=\text{div}\left[\left(1-\frac{1}{\left|\nabla \phi \right|}\right)\nabla \phi \right]$$

which has the factor (1-1/| ∇ *ϕ*|) as the diffusion rate. If |∇ *ϕ* |>1, the diffusion rate is positive and the diffusion affects *ϕ*, and therefore closer to the desirable property |∇ *ϕ* |=1 of SDF. If |∇ *ϕ* |<1, the term acts as a reverse diffusion.

A classic iterative process to minimize the functional *E* is the following gradient flow with an artificial time *t*:

(14) $$\left\{ \begin{array}{l} \phi_{\left(t=0\right)}=\phi_{0}\\ \frac{\partial \phi }{\partial t}=Q(\phi ) \end{array}\right.$$

where *ϕ*_0_ is the initial level set function. The means *c*_1_ and *c*_2_ of regions *w*_k+1_ and *w̅*_k+1_ are calculated from the mean of intensity values on image *I* with restriction of level set function *ϕ* on the sub-region *w_k_* and expressed by:

(15) $$c_{1}(\phi )=\frac{\int_{\Omega }I(x)H(-\phi (x))M^{k}(x)dx}{\int_{\Omega }H(-\phi (x))M^{k}(x)dx}$$

(16) $$c_{2}(\phi )=\frac{\int_{\Omega }I(x)H(\phi (x))M^{k}(x)dx}{\int_{\Omega }H(\phi (x))M^{k}(x)dx}$$

If one of the sub-regions *w_k_*_+1_ or *w̅_k_*_+1_ is empty, then the formulation degenerates, and therefore the algorithm automatically terminates. Finally, the principal steps of the algorithm are:

Initialize *k=*0
Compute *M^k^* by Equation (7) and initialize *ϕ* by *ϕ*_0_
Solve the PDE for *ϕ* with Equation (14) to obtain *φ^k^*^+1^
Check whether the solution is stationary. If not, *k*=*k*+1 and repeat from step 2

In this paper, we note that the stationary problem obtained directly from the minimization problem could also be solved numerically using a similar finite difference scheme.

## 4. Experimental Implementation Section

### 4.1. De-Noising Image Field

Medical images are affected by artifacts due to device noise and inhomogeneities in the body. The noise affects the segmentation process. The noise changes the mean of a region and behaves like a strong edge. We use an image de-noising technique to reduce the interference of noise. In Rudin, Osher, and Fatemi (ROF) [15] their famous denoising model was based on total variation minimization and PDE. The ROF model preserves the edge while removing the noise. Let Ω be an open set representing the image domain and *I_0_* be a given image. The denoised image *I* minimizes:

(17) $$E_{\text{ROF}}(I;\lambda )=\int_{\Omega }|\nabla I|dx+\lambda \int_{\Omega }{\left|I_{0}-I\right|}^{2}dx$$

where a parameter λ > 0 controls the balance of minimization between the spatial change term on *I* and difference term to *I*_0_. We diffuse the *I*_0_ by changing λ. The denoised image *I* is obtained using the gradient decent with the Euler-Lagrange equation, which is the minimization condition of Equation (17).

### 4.2. Numerical Scheme

In our work, the Dirac function *δ*(*z*) and the Heaviside function *H*(*z*) used in Equations (12), (15) and (16) are the smoothed versions of the Dirac function and the Heaviside function over the entire region. The approximations *H*_ε_(*z*) and δ_ε_(*z*), as proposed in [6], are:

(18) $$H_{ɛ}(z)=\frac{1}{2}\left(1+\frac{2}{\pi }\arctan\left(\frac{z}{ɛ}\right)\right)$$

(19) $$\delta_{ɛ}(z)=\frac{ɛ}{\pi (z^{2}+{ɛ}^{2})}$$

We use the regularized Dirac δ_ε_(*z*) and the Heaviside *H_ε_* (z) with ε = 1.5 for all the experiments in this paper. All the spatial partial derivatives *dϕ / dx* and *dϕ / dy* are approximated by forward, backward, and central differences. The characteristics of the term indicates whether to use a forward, backward, or central difference method should be used for *ϕ*. In numerical implementation, we try to use each element of *ϕ* equally. The forward difference $D_{x}^{+},D_{y}^{+}$, the backward difference $D_{x}^{-},D_{y}^{-}$ and the central difference $D_{x}^{c},D_{y}^{c}$ for *ϕ* can be computed as follows:

(20) $$\begin{array}{ll} D_{x}^{+}\phi_{i,j}=\phi_{i+1,j}-\phi_{i,j}, & D_{y}^{+}\phi_{i,j}=\phi_{i,j+1}-\phi_{i,j}\\ D_{x}^{-}\phi_{i,j}=\phi_{i,j}-\phi_{i-1,j}, & D_{y}^{-}\phi_{i,j}=\phi_{i,j}-\phi_{i,j-1}\\ D_{x}^{c}\phi_{i,j}=\frac{\phi_{i+1,j}-\phi_{i-1,j}}{2}, & D_{y}^{c}\phi_{i,j}=\frac{\phi_{i,j+1}-\phi_{i,j-1}}{2} \end{array}$$

where the indices of *ϕ* denote the coordinate of the image domain. Computing *x* and *y* components *N_x_* and *N_y_* of term ∇ *ϕ* /|∇ *ϕ* |= { *N_x_*,*N_y_*} is shown below:

(21) $$N_{x}=\frac{D_{x}\phi }{\sqrt{{\left(D_{x}\phi \right)}^{2}+{\left(D_{y}\phi \right)}^{2}+{ɛ}_{d}}},N_{y}=\frac{D_{y}\phi }{\sqrt{{\left(D_{x}\phi \right)}^{2}+{\left(D_{y}\phi \right)}^{2}+{ɛ}_{d}}}$$

where ε*_d_* is a small constant value to prevent dividing-by-zero and *D_x_*, *D_y_* are difference operators. The divergence of ∇ *ϕ* /|∇ *ϕ* | in Equation (12) is approximated by:

(22) $$\begin{array}{cc} \text{div}\left(\frac{\nabla \phi }{\left|\nabla \phi \right|}\right) & ≅\frac{\partial N_{x}}{\partial x}+\frac{\partial N_{y}}{\partial y}\\ & ≅D_{x}^{-}\left(\frac{D_{x}^{+}\phi }{\sqrt{{\left(D_{x}^{+}\phi \right)}^{2}+{\left(D_{y}^{c}\phi \right)}^{2}+{ɛ}_{d}}}\right)+D_{y}^{-}\left(\frac{D_{y}^{+}\phi }{\sqrt{{\left(D_{x}^{c}\phi \right)}^{2}+{\left(D_{y}^{+}\phi \right)}^{2}+{ɛ}_{d}}}\right) \end{array}$$

The Laplacian operator has been implemented in a similar manner.

### 4.3. Selection of Time Steps and Other Constants

In our experiments, we choose the following parameters: λ_1_= λ_2_=100, *μ*=200/225^2^, *v*=0, α =0.2/ *τ*, *τ* =1 where *τ* is a time step in the numerical implementation of Equation (14). We know that the time step *τ* and the coefficient α must satisfy *τ*α <1/4 to maintain stable level set evolution [5]. Using a larger time step can speed up the curve evolution, but may cause errors in the boundary location if the time step chosen is too large. There is a tradeoff between choosing a larger time step and accuracy in boundary location. In our case, we use *τ*α =0.2.

### 4.4. Initialization of Level Set Function

In outmoded level set methods, it is essential to initialize the level set function *ϕ* as an SDF *ϕ*_0_. If the initial level set function is expressively different from a signed distance function, then the re-initialization schemes are not able to re-initialize the function to a signed distance function. In our formulation, not only is the re-initialization procedure eliminated, but the level set function *ϕ* also no longer requires initialization as a signed distance function by the penalizing energy in [5]. We propose the following functions as the initial level set function *ϕ_0_*, where the denoised image *I*(*x*) and *M^k^* (*x*) is the calculated value of mask for every calculated *φ^k^*^+1^ value, that is, for every evolved level set function. Performing from the lowest intensity region, the initial level set function *ϕ*_0_is defined as:

(23) $$\phi_{0}(x)=\left\{ \begin{array}{ll} +\rho & \text{for}\;M^{k}(x)=0\;\text{or}\;I(x)<\min\left\{I(x)|M^{k}(x)=1\right\}+\kappa \\ -\rho & \text{otherwise} \end{array}\right.$$

where ρ > 0 is a constant and κ > 0 is a small constant. In this section, we use *ϕ*_0_ with ρ =4ε and κ = 1. By definition, this initial level set function *ϕ_0_* takes only two values: −4ε and 4ε.

## 5. Results and Discussion

The proposed multi-phase segmentation algorithm has been applied to synthetic and real mammographic images from the mini-MIAS database [16]; the range of intensity in all images is represented from 0 to 255 and the images are 1,024 × 1,024 pixels in size. We show the approximated circle enclosing the abnormality from the database, and the two-phase segmentations in each pass *k* of Equation (3) with a number of iterations of Equation (14).

**Figure 4.**
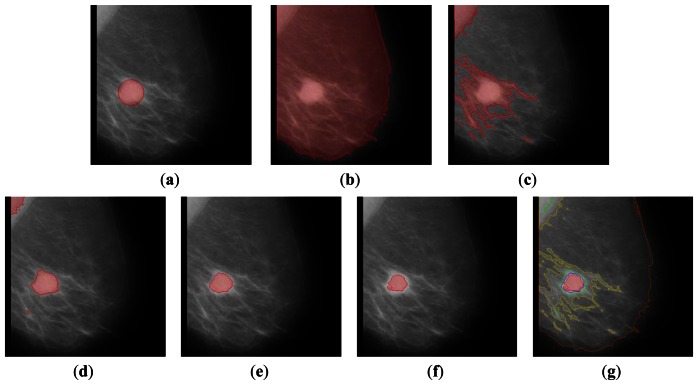
The mini-MIAS database of mammogram number 028. (**a**) The approximated region of abnormality from the database. (**b**) Contour 1 after 9 iterations of (14). (**c**) Contour 2 after 7 iterations. (**d**) Contour 3 after 7 iterations. (**e**) Contour 4 after 12 iterations. (**f**) Last contour 5 after 17 iterations. (**g**) Final contour map.

**Figure 5.**
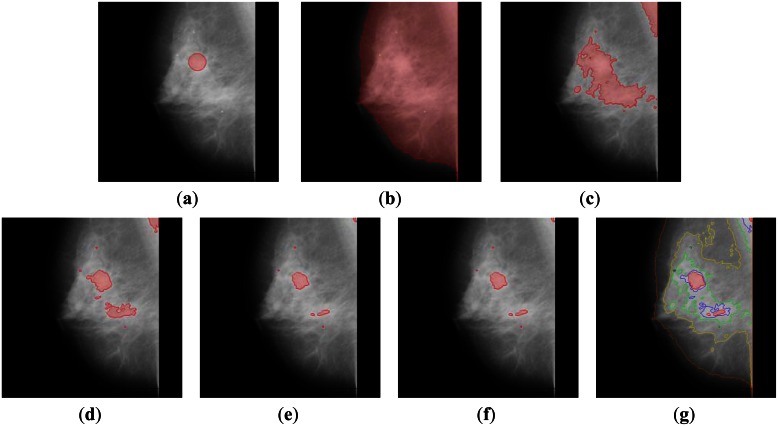
The mini-MIAS database of mammogram number 063 (**a**) The approximated region of abnormality from the database. (**b**) Contour 1 after 8 iterations of (14). (**c**) Contour 2 after 12 iterations. (**d**) Contour 3 after 9 iterations. (**e**) Contour 4 after 11 iterations. (**f**) Last contour 5 after 12 iterations. (**g**) Final contour map.

In Figures 4 and 5, we illustrate the result of real mammographic images and the approximated region of abnormality from the database. As represented, the proposed algorithm partitions the images, including weak and blurred edges. The recursive segmentation on the higher intensity region finely segments the region. In Figure 5, we consider a more difficult case, which is an abnormality region in a high intensity region. Our algorithm reduces the number of contours in the map from an average of 206 contours to 11 contours.

**Figure 6.**
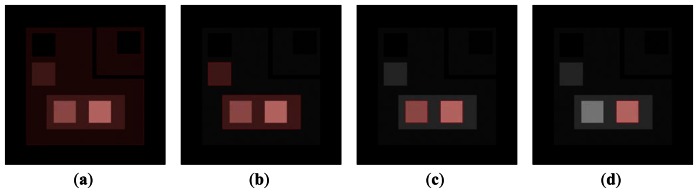
Results of a synthetic image with 5% uniform noise. (**a**) Contour 1 after 3 iterations of (14). (**b**) Contour 2 after 7 iterations. (**c**) Contour 3 after 7 iterations. (**d**) Contour 4 after 7 iterations.

Figure 6 shows the segmentation results of each pass on a synthetic image with 5% uniform noise. The red regions represent the inside partition, and the other regions indicate the outside partition. Each two-phase segmentation pass is converged after a number of iterations of Equation (14). The proposed algorithm works well on sharp-edged objects.

## 4. Conclusions

This paper presents an adaptive contour map that captures topographic information in mammograms characterized by blurred object boundaries. The proposed multipass active contour algorithm for adaptive contour mapping is based on the two-phase piecewise constant segmentation model (ACWE) proposed by Chan and Vese [6] and the variational level set formulation of curve evolution without re-initialization (LSEWR) proposed by Li *et al* [5]. Our algorithm performs spatial segmentation iteratively to find the optimal contours. It starts with the initial contour selected in the darkest region of a mammographic image so that the brighter regions of the image can be segmented and analyzed in a more refined way at later stages. The proposed algorithm provides an optimized topographic representation of mammograms that can increase the computational efficiency and accuracy of the analysis.

Our algorithm produces an arbitrary number of regions, and it automatically terminates when its stopping condition is met. The proposed algorithm was tested using synthetic and real mammographic images that include masses varying in size and subtlety. The experimental results showed that our approach yields an accurate contour map of both distinctive and subtle masses in mammograms. It also successfully produced an adaptive contour map in synthetic images that have relatively clear edges. The experimental results show sensitive segmentation on the important region as well as intuitive segmentation structure.
